# Production of Human Acid-Alpha Glucosidase With a Paucimannose Structure by Glycoengineered *Arabidopsis* Cell Culture

**DOI:** 10.3389/fpls.2021.703020

**Published:** 2021-07-14

**Authors:** Ratna Sariyatun, Florence, Hiroyuki Kajiura, Takao Ohashi, Ryo Misaki, Kazuhito Fujiyama

**Affiliations:** ^1^Laboratory of Applied Microbiology, International Center for Biotechnology, Osaka University, Suita, Japan; ^2^Institute for Open and Transdisciplinary Research Initiatives (OTRI), Osaka University, Suita, Japan; ^3^Cooperative Research Station in Southeast Asia (OU:CRS), Faculty of Science, Mahidol University, Bangkok, Thailand

**Keywords:** acid-alpha glucosidase, α1,3 mannosyltransferase, *alg3*, GAA, paucimannose, Pompe disease

## Abstract

Plant cell cultures have emerged as a promising platform for the production of biopharmaceutics due to their cost-effectiveness, safety, ability to control the cultivation, and secrete products into culture medium. However, the use of this platform is hindered by the generation of plant-specific *N*-glycans, the inability to produce essential *N*-glycans for cellular delivery of biopharmaceutics, and low productivity. In this study, an alternative acid-alpha glucosidase (GAA) for enzyme replacement therapy of Pompe disease was produced in a glycoengineered *Arabidopsis alg3* cell culture. The *N*-glycan composition of the GAA consisted of a predominantly paucimannosidic structure, Man_3_GlcNAc_2_ (M3), without the plant-specific *N*-glycans. Supplementing the culture medium with NaCl to a final concentration of 50 mM successfully increased GAA production by 3.8-fold. GAA from an NaCl-supplemented culture showed a similar *N*-glycan profile, indicating that the NaCl supplementation did not affect *N*-glycosylation. The results of this study highlight the feasibility of using a glycoengineered plant cell culture to produce recombinant proteins for which M3 or mannose receptor-mediated delivery is desired.

## Introduction

The market of biopharmaceutical proteins has been a rapidly developing area of economics. Accordingly, large biotechnological and pharmaceutical companies, such as Medicago, Ventria, Pfizer, Greenovation, and Epicyte, have a strong interest and large investment in the development of novel platforms for producing pharmaceutical proteins (Zagorskaya and Deineko, 2017). In this environment, plant cell cultures have emerged as a potential bioproduction system for recombinant pharmaceuticals due to their greater cost-effectiveness and safety over other eukaryotic platforms (Hellwig et al., 2004; Xu et al., 2011; Santos et al., 2016). Plant cells can grow in simple medium, do not harbor any known human pathogens or bacterial endotoxins, and can conduct post-translational modifications similar to those in mammals (Moustafa et al., 2016; Zhang et al., 2016). Plant culture systems also share many similarities to microbial and mammalian cells, such as enabling a contained, controlled, and sterile production environment meeting the criteria of good manufacturing practice for producing biopharmaceutical proteins (Santos et al., 2016). Plant cell cultures have also shown a good track record for production of biopharmaceutics as shown by the commercialization and the US Food and Drugs Administration approval to carrot cell-produced β-glucocerebrosidase (Elelyso) for the treatment of Gaucher disease. Notably, plant media do not contain animal-derived products, and thus, they can be used for the development of halal pharmaceutics for Islamic people, which is emerging as a prominent global market with an industry worth USD 2.3 trillion (Yusuf and Yajid, 2016). Another advantage of using plant culture is that the product can be secreted into the plant culture medium, which generally contains mainly sucrose and salts and no macromolecules (Kwon et al., 2003), thereby simplifying the downstream purification step. Due to these features, plant cell cultures have attracted enormous attention as a next-generation platform for producing biopharmaceutics, with the potential to challenge other well-established production systems (e.g., microbial and mammalian cells).

*N*-Glycosylation is crucial for manufacturing biopharmaceutical glycoproteins, as it greatly affects the stability, activity, and pharmacodynamics of glycoproteins in the human body (Seeberger and Cummings, 2015). Therefore, it is not surprising that control over the *N*-glycan profile is a regulatory prerequisite for recombinant protein therapeutics prior to use in patients (Rudge and Nims, 2018). For this reason, differences between human and plant *N*-glycosylation have become a limiting factor in the use of plants for the production of pharmaceutics. Plants lack core α1,6-fucose, β1,4-galactose, and sialic acid residues on *N*-glycans. Instead, plants generate β1,2-xylose and core α1,3-fucose residues on *N*-glycans, which are absent in humans, thus raising a concern of potential immunogenic reaction upon parenteral administration of the plant-made pharmaceuticals to humans (Gomord et al., 2010). Moreover, the production of pharmaceutics with minimal glycan heterogeneity is highly desirable in order to obtain consistent efficacy, which remains a bottleneck even in the well-established mammalian cell expression system (Goochee et al., 1991; Sethuraman and Stadheim, 2006). Thus, it is essential to generate human-type and homogenous *N*-glycosylation in the plant expression system.

A number of glycoengineering strategies have been done to optimize the use of plant cells for producing biopharmaceutical glycoproteins for human therapy. The main approaches consist of (1) elimination of the potentially immunogenic plant-specific *N*-glycans and (2) introduction of human-type glycosyltransferases into the plant cell system (Strasser et al., 2014; Montero-Morales and Steinkellner, 2018). The former can be done by cellular targeting of recombinant proteins into the ER by fusion with KDEL/SEKDEL ER retention signal or by knocking-out/down the plant β1,2-xylosyltransferases, α1,3-fucosyltransferases, and *N*-acetylglucosaminyltransferase-I enzymes. On the other hand, the introduction of human-type glycosyltransferases has been done by co-expressions of the human *N*-acetylglucosaminyltransferases-IV and V, galactosyltransferase, and sialic-acid synthesizing enzymes (Strasser et al., 2014; Montero-Morales and Steinkellner, 2018; Rozov et al., 2018). Surprisingly, introductions of these genes do not affect the plant phenotype, indicating that plant cells harbor high plasticity to tolerate multiple human-type glycosyltransferase enzymes (Strasser et al., 2014).

Certain *N*-glycan structures are often required to mediate delivery of biopharmaceutics to the target cells. Mannose-terminal *N-*glycans are capable of directing cellular delivery of biopharmaceutics *via* binding with mannose receptor (MR) expressed on the cell surface. Notably, the number of exposed terminal mannose residues has been shown to affect the efficacy of MR-mediated delivery. The paucimannosidic structure Man_3_GlcNAc_2_ (M3; Man: mannose; and GlcNAc: *N*-acetylglucosamine) exhibits a higher level of binding with MR than other *N*-glycans bearing more mannose-terminal residues/high-mannose structures (e.g., M5-M9; Van Patten et al., 2007; Shen et al., 2016). Moreover, M3 also has a clearance rate in mammalian hosts that is lower than those of high-mannose structures due to its lower affinity for binding with mannose-binding lectin (Van Patten et al., 2007). Unfortunately, the M3 structure is scarcely found in plant glycoproteins (Kajiura et al., 2010). Thus, a glycoengineering strategy is required in order to optimize the use of plant cells for producing biopharmaceutics bearing the M3 structure for MR-mediated delivery.

Our previous study showed that a glycoengineered *Arabidopsis* plant lacking the activity of α1,3-mannosyltransferase (ALG3), a so called *alg3* mutant, resulted in the generation of a predominantly M3 structure and a lower amount of plant-specific *N*-glycans (Kajiura et al., 2010). These characteristics imply that this glycoengineered plant line would be of great benefit for producing biopharmaceutics with the M3 structure to mediate delivery through the MR pathway and lacking plant-specific *N*-glycans. To date, however, there have been no studies exploring the use of *Arabidopsis alg3* suspension cell culture for the production of biopharmaceutics.

In this study, an *Arabidopsis alg3* cell culture was developed and used for producing acid-alpha glucosidase (GAA). GAA is an enzyme that catalyzes the breakdown of glycogen into glucoses in the acidic milieu of the lysosome. In human cells, GAA is produced as 110 kDa precursor, 95 kDa intermediate, and 76/70 kDa mature glycoproteins (Moreland et al., 2005). Deficiency of this enzyme results in Pompe disease, which is characterized by lysosomal accumulation of glycogen leading to severe metabolic myopathy (Kohler et al., 2018). The advent of enzyme replacement therapy (ERT) by injection with recombinant human GAA has prolonged the life span of patients with Pompe disease by restoring the function of cardiac muscles. However, the ability of ERT to improve the manifestations related to skeletal muscle (e.g., motor weakness, speech difficulties, dysphagia, osteopenia, and macroglossia) and smooth muscle damages (e.g., respiratory, vascular, gastrointestinal, genitourinary, ocular, and dermatologic smooth muscle pathologies) has not fully met expectations (Kohler et al., 2018; McCall et al., 2018). The latter ability is critical because smooth muscle damages in the lower respiratory tract could contribute to respiratory failure, which is the main cause of death in patients with late-onset Pompe disease despite use of ERT (Meena and Raben, 2020). This failure of ERT to fully restore the functions of skeletal and smooth muscle cells is due to the low number of mannose-6-phosphate receptors on the cell surface of these cells, which results in insufficient enzyme delivery to the lysosome (Wenk et al., 1991). Importantly, MR is known to be expressed on smooth muscle cells (Lew et al., 1994; Taylor et al., 2005). By using an *Arabidopsis alg3* cell culture, it would be possible to produce an alternative GAA that bears the M3 structure and allows cellular delivery through MR, thereby outperforming the efficacy of the commercial GAA for targeting smooth muscle cells.

A common limitation of plant cultures is their low productivity (Su and Lee, 2007; Xu and Zhang, 2014). In plant cell cultures, medium or nutrient engineering is considered as important as the expression construct and cell line development for increasing the protein productivity (Holland et al., 2010; Xu et al., 2011; Fischer et al., 2015). However, because plant cultures are still relatively new platform compared to other well-established approaches for the production of biopharmaceutics, such as mammalian and microbial cells, there have been few investigations into methods for enhancing protein secretion into the plant culture media. Previous studies have demonstrated the effects of protein stabilizers [e.g., polyvinylpyrrolidone (PVP), polyethylene glycol (PEG), bovine serum albumin (BSA), NaCl, gelatin, and dimethyl sulfoxide (DMSO)] to improve recombinant protein production in plant cell cultures (LaCount et al., 1997; James et al., 2000; Sharp and Doran, 2001; Lee et al., 2002). Others have also shown enhanced recombinant protein production by using mannitol to induce osmotic stress (Tsoi and Doran, 2002) or modifying nitrogen content of the medium (Zhang et al., 2016). However, none has been tested in *Arabidopsis* or for GAA production.

In the present study, we develop an *Arabidopsis alg3* cell culture for the production of GAA bearing the M3 structure. The effects of medium engineering on the GAA production in *Arabidopsis* cell culture are also examined. The results of this study will contribute to optimization of plant cell cultures to produce recombinant proteins.

## Materials and Methods

### Plasmid Construction

A modified pGPTV-BAR binary plasmid containing the pAt-GC-HSP cassette was previously constructed in our laboratory (Limkul et al., 2015). To prepare it for use in GAA expression, this plasmid was amplified using the primers pFK1-Fw (5'-GCT ACT AGT TAT CAA CAT GAA GAA CTT GCT TTT G-3') and pFK1-Rv (5'-GGT ACC GAG CTC ATA TGA AGA TGA AG-3'; the underlined letters indicate the *Spe*I restriction site) to generate a linear fragment without the GC sequence and containing a *Spe*I site near the 5'-end. Another *Spe*I restriction enzyme site was added by using the primers Mut*Spe*I_Fw (5'-TCA TAT GAG CTC GGT ACC AGT CGA CTA GT-3') and Mut*Spe*I_Rv (5'-CGG TAC CAG TCG ACT AGT TAT CAA CAT GA-3'), generating the linear fragment pFK1-BAR. On the other hand, an insert of GAA cDNA (NCBI NM_000152.3) was amplified from human liver cDNA along with addition of *Spe*I sites using the primers GAA-*Spe*I-Fw (5'-GTC ACT AGT ACC ATG GGA GTG AGG CAC CCG CC-3') and GAA-*Spe*I-Rv (5'-GAT ACA CTA GTC TAA CAC CAG CTG ACG AGA AAC TGC TCT CCC A-3'). Both the GAA and pFK1-BAR fragments were digested by *Spe*I restriction enzyme (New England Biolabs) and subsequently ligated using Ligation High Ver. 2.0 (Toyobo). This resulted in a circular binary plasmid containing the GAA expression cassette, which was designated pFK1-BAR-GAA.

### Plant Cell Culture and Generation of GAA-Producing *Arabidopsis alg3* Cell Culture

The *Arabidopsis alg3* cell culture was developed from leaves of our *Arabidopsis alg3* plant (Kajiura et al., 2010) using a standard protocol (Sanchez-Serrano and Salinas, 2014). Briefly, the leaves were sterilized, sectioned, and transferred into sterile Murashige and Skoog (MS) medium (Murashige and Skoog, 1962) containing 0.3% gellan gum for callus induction. After about a month in the dark at 25°C, calli were transferred into sterile liquid MS medium and incubated in the dark at 25°C on a rotary shaker at 120 rpm until becoming a suspension cell culture. The culture was maintained in MS medium on a rotary shaker at 120 rpm in the dark at 25°C. About 10 ml of fresh cells was inoculated into 100 ml of fresh MS medium weekly for maintenance.

The *Arabidopsis alg3* cell culture was transformed by the *Agrobacterium*-mediated transformation as follows. First, the binary plant expression vector pFK1-BAR-GAA was introduced into *Agrobacterium tumefaciens* LBA4404 cells by electroporation. The transformed *Agrobacterium* cells were cultivated in 2 × YT liquid medium containing kanamycin (50 μg/ml), streptomycin (100 μg/ml), and rifampicin (10 μg/ml) until an OD_600_ of 0.8. About 5 ml of 4-day-old *Arabidopsis* cells was mixed with 200 μl of *Agrobacterium* solution on a petri dish and incubated in the dark at 25°C for 2–3 days. Subsequently, cells were washed with MS liquid medium containing 250 mg/l carbenicillin before being selected on MS medium containing bialaphos (10 μg/ml). Successfully transformed *Arabidopsis* calli were checked by Western blot to confirm the GAA production. Calli were grown in one whole petri dish and subsequently propagated in 100 ml of MS liquid medium in the dark at 25°C on a rotary shaker at 120 rpm speed until becoming a suspension cell culture. For maintenance, about 10 ml of fresh cells was transferred into 100 ml of fresh MS liquid medium weekly.

### Protein Extraction and Western Blot Analysis

Harvested cells were dissolved in lysis buffer (20 mM Tris-HCl pH 7.5, 10 mM EDTA, and 1 ml/mg of cells). The mixture was sonicated for 1 min and centrifuged at 12,000 × *g* for 20 min at 4°C, and then the supernatant was collected as the intracellular protein extract. Meanwhile, analysis of secreted proteins was conducted using the harvested culture media directly. Protein concentration of samples was determined by Bradford assay.

The extracted intracellular and extracellular proteins were separated *via* sodium dodecyl sulfate-polyacrylamide gel electrophoresis (SDS-PAGE). The separated proteins were transferred into a nitrocellulose membrane in transfer buffer (50 mM Tris, 40 mM glycine, and 20% methanol) using a mini-trans blot apparatus (Bio-Rad) for 90 min. To prevent non-specific binding, membranes were blocked with 5% non-fat milk powder in phosphate-buffered saline containing 0.05% Tween 20 buffer with gentle agitation on a rotary shaker at 20 rpm for 1 h. The membrane was incubated with a 1:5,000 dilution of anti-GAA monoclonal antibody produced in rabbit (Abcam) or 1:10,000 dilution of anti-horseradish peroxidase (HRP) polyclonal antibody produced in rabbit (Sigma-Aldrich) for 1 h, followed by a 1:5,000 dilution of anti-rabbit IgG conjugated to alkaline phosphatase (Sigma-Aldrich) for 1 h. For detection, Luminata^™^ Forte Western HRP substrate (Merck Millipore) was added to the membrane, and luminescent signals on the membrane were detected using the Invitrogen iBright Imaging System 1,500 Series (Thermo Fisher Scientific).

### GAA Enzyme Activity Assay

GAA activity was measured by hydrolysis of the synthetic substrate *p*-nitrophenyl α-D-glucopyranoside (Santa Cruz Biotechnologies) in 50 mM sodium acetate pH 4.3 containing 0.1% BSA. The assay was performed at 37°C for 30–60 min and stopped by the addition of 0.87 M sodium bicarbonate pH 11.0. Hydrolysis of the substrate was monitored by release of the reaction product, *p-*nitrophenol, which can be measured at an absorbance of 405 nm by using iMark^™^ Microplate Reader (Bio-Rad). One unit (U) of activity was defined as the amount of activity that resulted in the hydrolysis of 1 μmol of substrate per minute at 37°C under the assay condition.

### GAA Purification

The medium of GAA-producing *Arabidopsis alg3* culture was filtered through a Glass Econo-Column (Bio-Rad). NaCl was subsequently added to the medium until reaching a final concentration of 4 M. Then, the NaCl-containing medium was loaded into a hydrophobic interaction chromatography column (Toyopearl Phenyl-650 M, Tosoh Corporation) pre-equilibrated in 20 mM Tris-HCl pH 7.5 with 4 M NaCl. After washing the column using 4 M NaCl in 20 mM Tris-HCl pH 7.5 buffer, GAA was eluted by decreasing NaCl concentration. GAA-containing fractions were dialyzed to exchange the buffer into 20 mM sodium acetate pH 4.3. The dialyzed sample was applied to a cation exchange chromatography column (Toyopearl SP 550C, Tosoh Corporation) pre-equilibrated in 20 mM sodium acetate pH 4.3 buffer. After washing the column, GAA was eluted with 20 mM sodium acetate pH 4.3 containing 0.1–0.5 M NaCl. GAA-containing fractions were concentrated using a Vivaspin 20 with a 10 kDa cutoff (Sartorius Stedim Biotech GmbH).

### Manipulation of Medium Composition

Polyvinylpyrrolidone (MW 360,000, 0.125 g/ml), PEG (MW 8,000, 0.2 g/ml), and BSA (10%) stock solutions were prepared and filter-sterilized using a Millex-GP syringe filter unit, 0.22 μm (Merck Millipore) before use, while 5 M NaCl, 0.5 M EDTA, and 1.0 M ammonium nitrate (NH_4_NO_3_) stock solutions were sterilized by autoclaving. Then, appropriate amounts of PVP, PEG, BSA, EDTA, and DMSO stock solutions were added to 100 ml of MS medium to final concentrations of 0.75 g/l, 2 g/l, 0.1%, 1 mM, and 4%, respectively, while NaCl was added to final concentrations of 25 mM, 50 mM, 100 mM, and 150 mM. Meanwhile, supplementation of gelatin 5% (w/v) and mannitol 34 g/l (186.6 mM) was conducted by inputting appropriate amounts of stock powders before autoclaving the MS medium. While the MS medium already contains NH_4_NO_3_ (165 mg/100 ml), in some experiments an additional 330 mg of NH_4_NO_3_ was added to the 100 ml of MS medium from the stock solution resulting in final NH_4_NO_3_ concentration of 495 mg/100 ml (61.8 mM).

In all experiments, 10 ml of fresh cells was inoculated into 100 ml of MS medium in 300 ml flasks. All of the tested compounds were added on the day of cell cultivation (day 0), and then, the cultures were grown for 14 days. One ml of cell culture was taken on each of days 0, 4, 7, 11, and 14 of treatment. The cells were separated from the medium for the determination of fresh cell weight and the analysis of intracellular proteins. The medium was also collected for the analysis of secreted proteins. All samples were stored at −20°C until analysis.

### Analysis of *N*-Glycan Structures Attached to GAA Produced in *Arabidopsis alg3* Cell Culture

Purified GAA was separated on SDS-PAGE and stained with Coomassie Brilliant Blue (CBB). The 95 and 76 kDa GAA bands were excised from the gel. The GAA-containing gels were sliced and destained with methanol:50 mM NH_4_HCO_3_ (1:1 v/v) for 2 min with intermittent vortex mixing followed by overnight destaining in acetonitrile:50 mM NH_4_HCO_3_ (1:1 v/v) with intermittent vortex mixing. Then, the GAAs were digested in the gel using Trypsin Gold (Promega) in ProteaseMAX^™^ Surfactant (Promega) at 50°C for 1 h. The reaction was terminated by addition of trifluoroacetic acid to a final concentration of 0.5%. Digested glycopeptides were extracted from the gel, dried, and dissolved in 0.1% formic acid prior to injection into a nanoLC-MS/MS system. The nanoLC-MS/MS analysis was performed on an ESI-Qq-TOF mass spectrometer (micrOTOF-Q II; Bruker Daltonics) using a nanoLC system (1,200 series; Agilent Technologies) incorporating a trap column (5 μm, 0.3 × 5 mm) and analytical column (3.5 μm, 0.075 × 150 mm), both packed with Zorbax 300SB C-18 (Agilent Technologies). For the nanoLC system, the mobile phase consisted of 0.1% formic acid in water (solvent A) and 0.1% formic acid in acetonitrile (solvent B). The tryptic peptides were trapped in the column at a flow rate of 10 μl/min for 5 min. Elution was performed at a flow rate of 0.6 μl/min using a 2 to 8% gradient of solvent B over 5 min followed by a linear increase of solvent B to 50% for 40 min at 35°C. After elution, the column was washed with 95% solvent B for 5 min before returning to the initial conditions. For MS and MS/MS analyses, the system was operated with automatic switching between MS and MS/MS modes. The operating parameters were set as follows: positive-ion mode, mass range 50–4,500 *m/z*, nebulizer flow 1.0 psi, dry gas flow rate 5.0 l/min, dry temperature 180°C, and ISCID energy 5.0 eV. The three most abundant signals (absolute threshold >20 counts/s) were selected on each MS spectrum for further isolation and fragmentation. The complete system was fully controlled by micrOTOF control software (Bruker Daltonics). Bruker Compass DataAnalysis (version 4.0) was used for deconvolution of MS spectra and glycan analysis, and BioTools (version 3.2) was used for *de novo* sequencing.

## Results

### GAA Production in the Transformed *Arabidopsis alg3* Cell Culture

**Figure 1:**
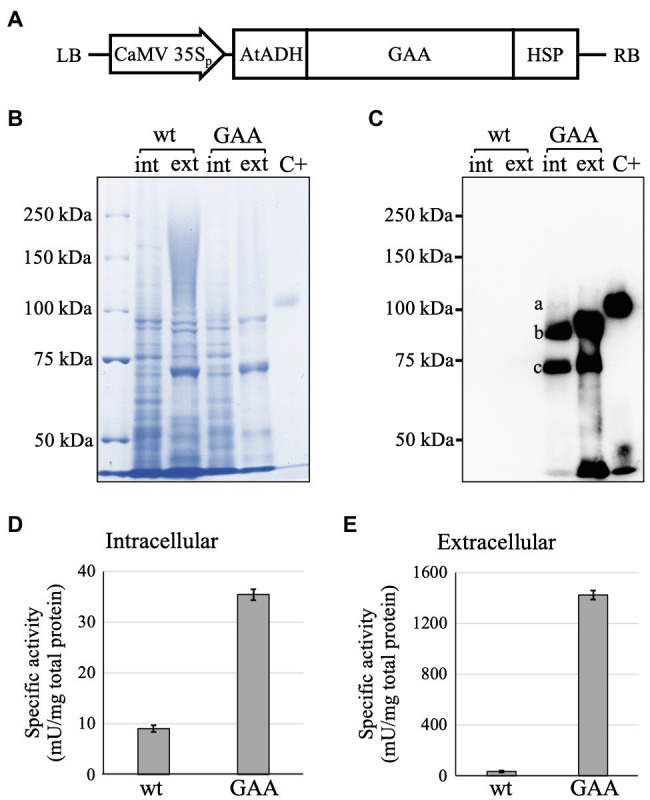
Expression cassette and confirmation of acid-alpha glucosidase (GAA) production in the transformed *Arabidopsis alg3* cell culture. **(A)** Schematic representation of pFK1-BAR-GAA. *Cauliflower mosaic virus* (CaMV) 35S_p_: CaMV 35S promoter; AtADH 5’-UTR: 5'-untranslated region of *Arabidopsis alcohol dehydrogenase* gene which acts as an enhancer; GAA: cDNA of human GAA; HSP: *Arabidopsis* heat shock protein terminator; and LB and RB: left and right borders of T-DNA, respectively. **(B,C)** CBB staining and Western blot analysis of untransformed (wt) and transformed (GAA) 7-day-old *Arabidopsis alg3* cell culture to confirm GAA production intra (int)- and extracellularly (ext). Marks a, b, and c indicate the 110, 95, and 76 kDa GAAs, respectively. C+ is human embryonic kidney/HEK293-derived recombinant human GAA used as a positive control. **(D,E)** GAA-specific activities in crude intracellular and extracellular proteins of untransformed (wt) and transformed (GAA) *Arabidopsis alg3* cell culture.

A GAA expression cassette was successfully constructed within pFK1-BAR-GAA. The cassette had a GAA sequence downstream of the *Cauliflower mosaic virus* (CaMV) CaMV 35S promoter and 5'-UTR AtADH enhancer, followed by an HSP terminator (Figure 1A). The binary plasmid was successfully inserted into *A. tumefaciens* LBA4404 cells, and then, the *Agrobacterium* was used for *Agrobacterium*-mediated transformation to generate GAA-producing *Arabidopsis alg3* cells. Putative transformants were selected with bialaphos. The GAA-producing calli were selected and used to establish a suspension cell culture. GAA production in the culture was confirmed by Western blot (Figures 1B,C). In human cells, GAA polypeptide is processed through sequential protease cleavages in the *N*- and *C*-termini generating 110 kDa GAA precursor, 95 kDa GAA intermediate, and 76 and 70 kDa GAA mature forms (Moreland et al., 2005). In *Arabidopsis alg3* cell culture, GAA was produced in the intracellular fraction as three forms corresponding to the 110, 95, and 76 kDa GAAs. The latter seemed to have slightly lower size than 76 kDa, which could be due to the different *N*-glycosylation from that in humans. Moreover, the 95 and 76 kDa GAAs were also secreted into the plant culture medium. Detection of these proteins indicated that the plant cells were capable of conducting a GAA processing mechanism similar to that in humans.

The GAA-producing *Arabidopsis* culture showed higher GAA-specific activity compared to an untransformed culture both intracellularly (9.1 ± 0.7 mU/mg in the *alg3* culture and 35.1 ± 5.2 mU/mg in the GAA-producing culture; Figure 1D) and extracellularly (35.4 ± 1.0 mU/mg in the *alg3* culture and 1,420 ± 37 mU/mg in the GAA-producing culture; Figure 1E). These results indicated that the *Arabidopsis*-produced GAA was functional. Notably, the extracellular fraction showed higher activity than the intracellular fraction, suggesting that most of the GAAs were secreted to the medium.

### GAA Purification

**Figure 2:**
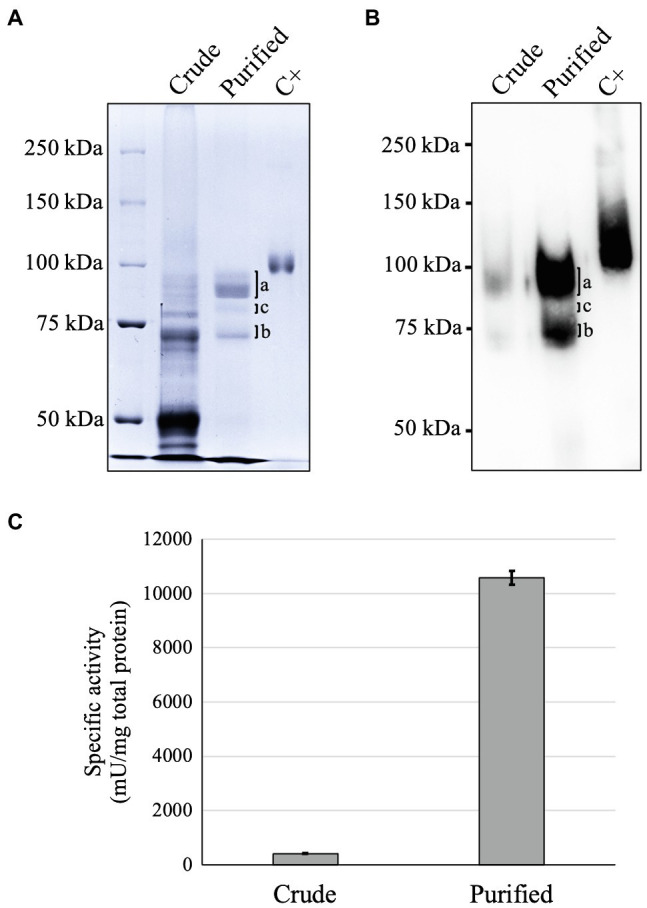
Analysis of GAA purified from GAA-producing *Arabidopsis alg3* cell culture by Coomassie Brilliant Blue (CBB) staining **(A)**, Western blot **(B)**, and GAA activity assay **(C)**. Marks a and b indicate the 95 and 76 kDa GAAs, respectively, whereas mark c indicates a degraded GAA (Supplementary Figure 1 and Supplementary Table 1). C+ is human embryonic kidney/HEK293-derived recombinant human GAA used as a positive control.

GAA was successfully purified from the plant culture medium using two-step chromatography columns. GAA was purified as 95 and 76 kDa forms (Figures 2A,B). The 95 kDa GAA contains seven *N*-glycosylation sites (N140, N233, N390, N470, N652, N882, and N925), whereas the 76 kDa GAA contains five *N*-glycosylation sites (N140, N233, N390, N470, and N652). Therefore, it was expected that some bands would be observed around these sizes due to the different glycoforms on each of these sites. A faint band between the 95 and 76 kDa GAAs in the CBB staining was absent in the Western blot. This band was a degraded GAA as indicated by detection of the same GAA *N*-glycosylation sites from this band (Supplementary Figure 1; Supplementary Table 1). The degradation should have occurred near the *N*-terminus, thereby causing failed detection by the monoclonal anti-GAA antibody which binds an epitope near the *N*-terminus of GAA (within aa 150–250). After purification, the GAA-specific activity in the purified sample (10,600 ± 250 mU/mg) increased up to 26 times compared to that in the crude sample (409 ± 8.0 mU/mg; Figure 2C). The yield of GAA purified from the medium of the *Arabidopsis alg3* cell culture was 2.5% of the initial sample, or 60.9 ± 12.4 μg/l (Supplementary Table 2).

### Effects of PEG, PVP, BSA, EDTA, DMSO, Gelatin, Mannitol, NH_4_NO_3_, and NaCl Supplementations to the *Arabidopsis alg3* Cell Growth and GAA Production

**Figure 3:**
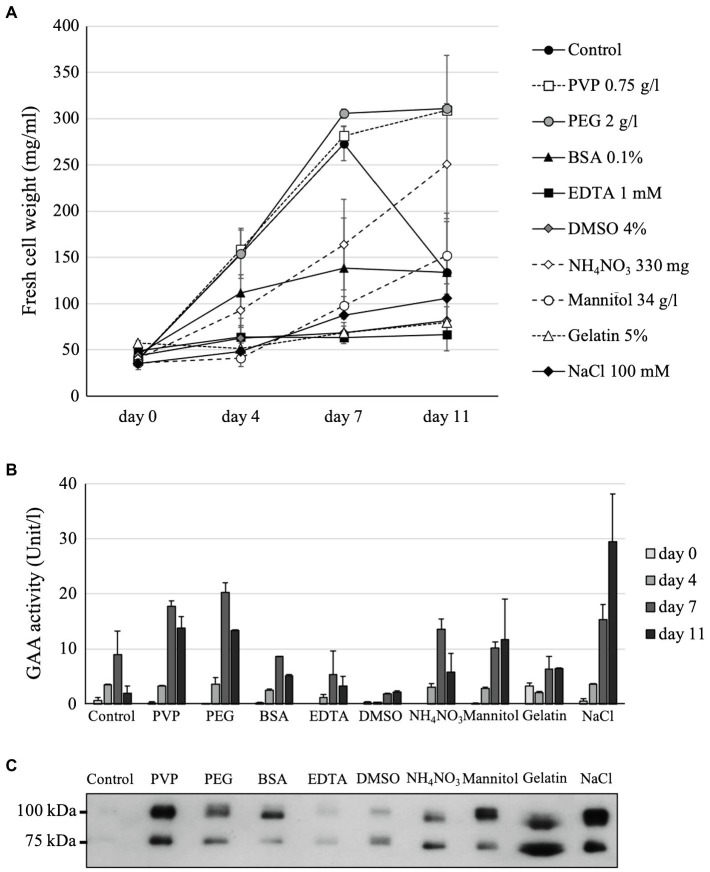
Effects of chemical additives on the cell growth of GAA-producing *Arabidopsis alg3* cell culture **(A)** as well as on GAA production as evaluated by a GAA activity assay **(B)**, and Western blotting **(C)**. Data of **(A)** are average of three independent replications. The media of 11-day-old cultures are used for the Western blot.

The following nine compounds were examined for their effects on the *Arabidopsis alg3* cell growth and GAA production: PVP, PEG, BSA, EDTA, DMSO, NH_4_NO_3_, mannitol, gelatin, and NaCl. These compounds have been reported to increase the production of some recombinant proteins in plant cell cultures. In the present study, the presence of 0.1% BSA, 4% DMSO, 34 g/l (186.6 mM) mannitol, 100 mM NaCl, 1 mM EDTA, or 5% gelatin in the MS medium severely inhibited the *Arabidopsis* cell growth. Conversely, addition of 0.75 g/l PVP or 2 g/l PEG increased the cell growth. Meanwhile, supplementing the MS medium with an additional 330 mg of NH_4_NO_3_ (61.8 mM) slightly decreased the cell growth, but lengthened the exponential growth phase (Figure 3A).

In comparison with the control, samples from the media supplemented with 0.75 g/l PVP, 2 g/l PEG, additional 330 mg (61.8 mM) NH_4_NO_3_, 34 g/l (186.6 mM) mannitol, or 100 mM NaCl showed higher GAA activities both at 7 and 11 days of culture. Those supplemented with 0.1% BSA or 5% gelatin showed higher activities than the control only at 11 days of culture. The highest GAA activity was observed at 11 days of culture supplemented with 100 mM NaCl (Figure 3B). These results were confirmed by Western blot analysis, which revealed GAA bands for the 11-day-old culture media (Figure 3C), except in the case of the gelatin-supplemented culture. Western blot analysis of the gelatin-supplemented culture showed thick band, indicating that gelatin helped stabilize the secreted GAA and prevent it from degradation. However, the activity assay showed low activity indicating that the presence of gelatin might hinder the assay. In addition, because gelatin supplementation severely inhibited the cell growth, caused medium solution to become viscous, and would be an additional contaminant protein in the purification step, we did not continue using it for further optimization. Instead, NaCl was chosen for further optimization to obtain the highest GAA production in the medium.

### Selection of an NaCl Concentration for Optimum GAA Production

**Figure 4:**
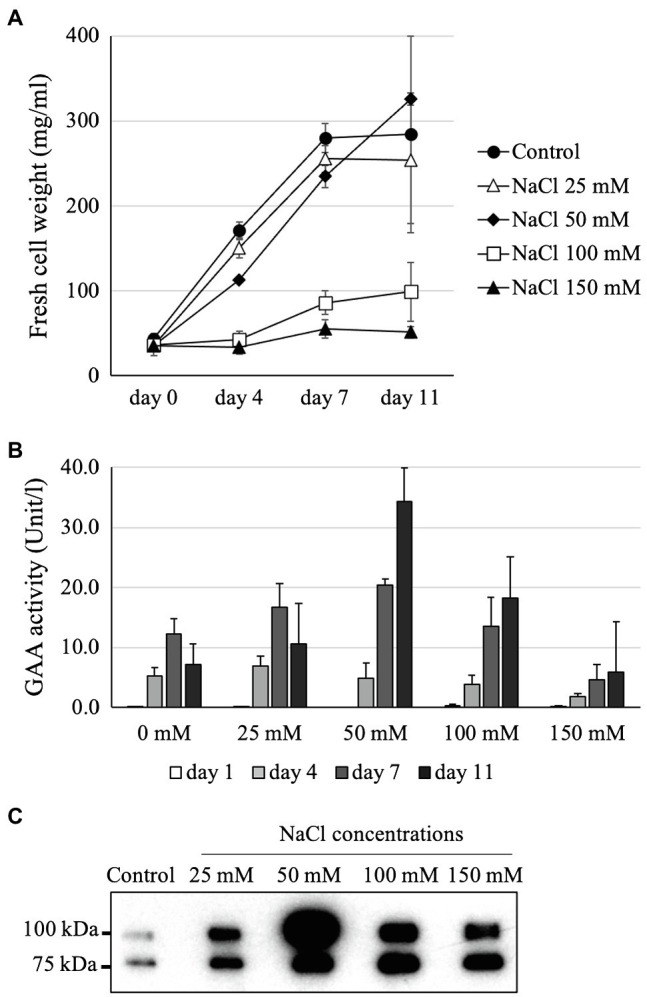
Optimization of the NaCl concentration for yielding the highest GAA production while maintaining *Arabidopsis alg3* cell growth. **(A)** Effects of addition of 0–150 mM NaCl on the cell growth of a GAA-producing *Arabidopsis alg3* cell culture. Data are average of three independent replications. **(B,C)** Evaluation of GAA accumulation in the NaCl-supplemented culture medium by GAA activity assay and Western blot, respectively. The media of 11-day-old cultures are used for the Western blot.

Supplementing the MS medium with 100 mM NaCl highly increased GAA accumulation in the medium, but also severely reduced the growth of GAA-producing *Arabidopsis* cells. Thus, further investigation was performed to determine the optimum NaCl concentration to obtain high GAA yield while maintaining cell growth. For this purpose, the GAA-producing *Arabidopsis alg3* cell culture was grown in MS media containing 0 mM (control), 25 mM, 50 mM, 100 mM, and 150 mM NaCl. The results showed that cell growth was severely inhibited under NaCl concentrations of 100 mM or more. The *Arabidopsis* cell culture maintained cell growth in the presence of 25 mM or 50 mM NaCl (Figure 4A). Among the tested groups, the highest GAA activity was observed in the medium sample from 11-day-old culture grown in MS containing 50 mM NaCl (Figure 4B). This result was confirmed by Western blot data showing the thickest band on this sample (Figure 4C).

**Figure 5:**
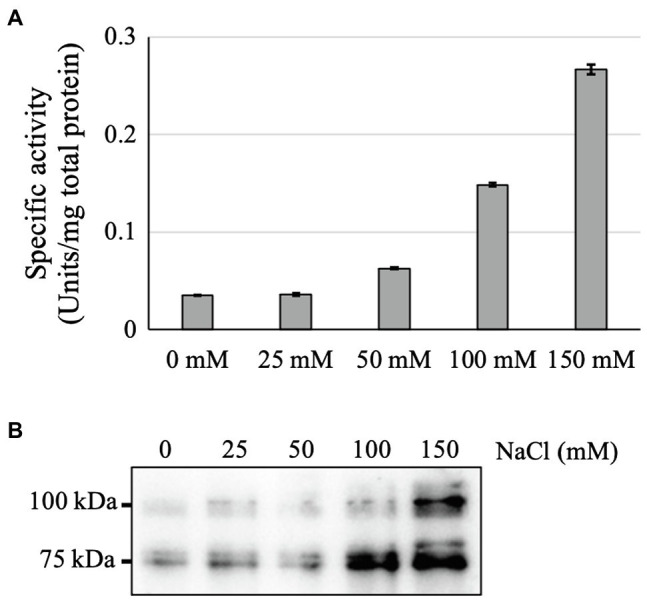
Effects of NaCl supplementation on intracellular GAA production. **(A)** Intracellular GAA-specific activity in cultures supplemented with various amounts of NaCl (0–150 mM). **(B)** Western blot of crude intracellular proteins of NaCl-supplemented 11-day-old culture cells. The result of CBB staining as a loading control is shown in Supplementary Figure 2.

To elucidate the reason for the higher GAA accumulation in the medium of NaCl-supplemented culture, the intracellular GAA production and activity were also tested. Interestingly, the intracellular GAA-specific activity and production were also increased with each increment of NaCl concentration (Figure 5). This suggested that the enhanced GAA accumulation in the medium was a result of increased intracellular GAA production. However, because the *Arabidopsis* cell growth was severely inhibited in the presence of 100 or 150 mM NaCl, the total GAA accumulation in the media of cultures supplemented with 100 or 150 mM NaCl was also low.

**Figure 6:**
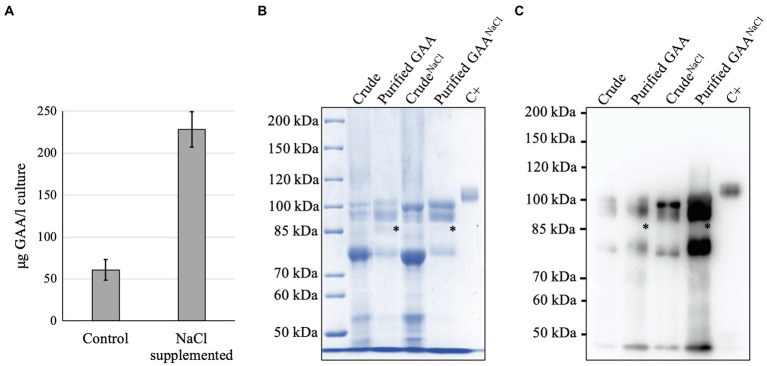
Analysis of GAA purification from NaCl-supplemented culture. **(A)** Yield of GAA production from cultures with and without NaCl supplementation. **(B,C)** CBB staining and Western blot of purified GAAs from cultures with and without NaCl supplementation. An asterisk (^*^) indicates degraded GAA.

After purification, the NaCl-supplemented culture resulted in 3.8-times higher yield of GAA production (228 ± 21.1 μg/l) than the control (60.9 ± 12.4 μg/l; Figure 6A). The GAA purity from NaCl-supplemented culture was also similar to that in the control (Figures 6B,C). Of note, the degraded GAA band that appeared between the 95 and 76 kDa GAAs was less visible in the purified GAA from the NaCl-supplemented culture. This indicated that NaCl helped protect GAA from degradation.

### *N*-Glycan Profiles of GAA Produced in *Arabidopsis alg3* Cell Culture With and Without NaCl Supplementation

**Figure 7:**
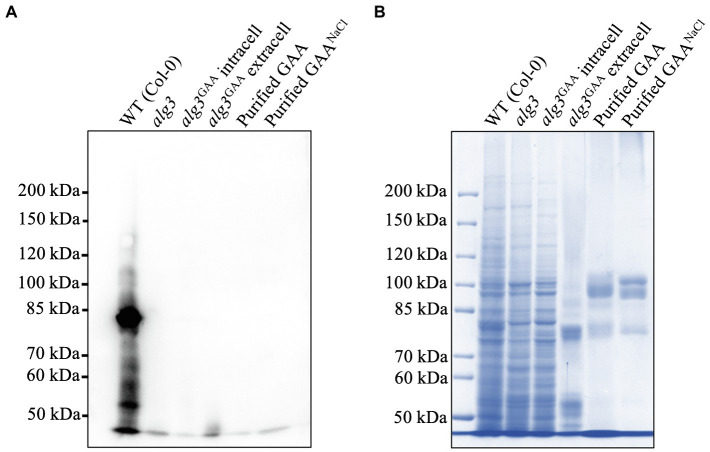
Detection of plant-specific *N*-glycans in an *Arabidopsis alg3* cell culture and purified GAA from the cultures with and without NaCl supplementation. **(A)** Western blot analysis using anti-HRP to detect plant specific *N*-glycans. **(B)** CBB staining as a loading control.

To identify the presence of plant-specific *N*-glycans, we performed Western blot analysis of crude and purified GAAs by using an anti-HRP antibody known to bind the plant-specific 𝝰1,3-fucose and β1,2-xylose residues (Laurière et al., 1989). The result showed that *Arabidopsis alg3* cell culture contained a small amount of plant-specific *N*-glycans (Figure 7). Notably, the Western blot data showed no signal in the sizes of GAA, suggesting that the GAA lacked the plant-specific *N*-glycans.

**Figure 8:**
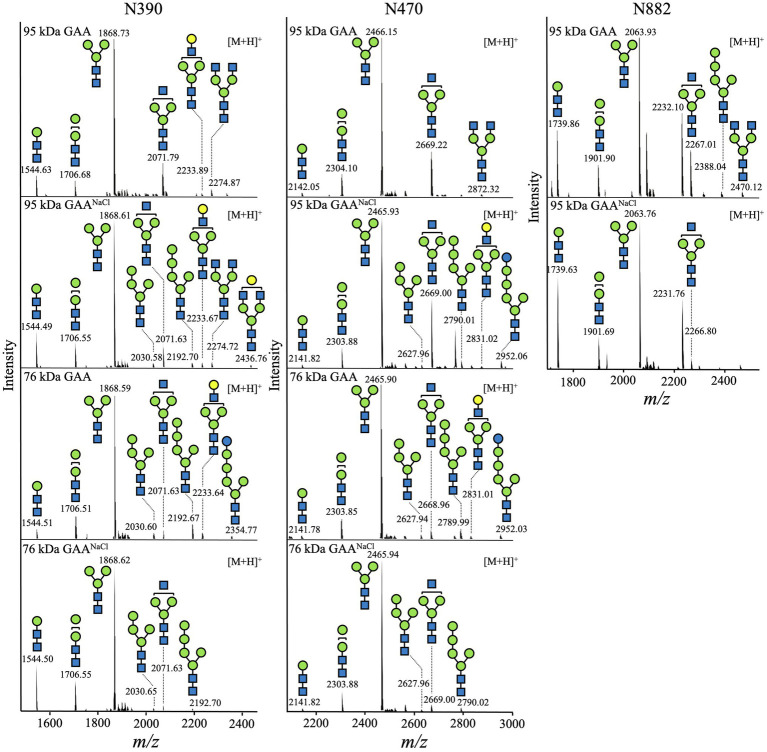
*N*-glycan profile of GAA produced in *Arabidopsis alg3* cell cultures with and without NaCl supplementation. In the shown deconvoluted MS spectra, the mass [M + H]^+^ of the tryptic glycopeptides carrying *N*-glycosylation sites (N390, N470, and N882) is depicted.

Constituent *N*-glycans of each *N*-glycosylation site in GAA were further determined by *de novo* sequencing of the tryptic GAA peptides using nanoLC-MS/MS analysis. The glycopeptides bearing the sequence of N390 (^386^QVVENMTR^393^), N470 (^465^GVFITNETGQPLIGK^479^), and N882 (^882^NNTIVNELVR^891^) were successfully detected. As expected, the majority of the *N*-glycans in both 95 and 76 kDa GAAs from *Arabidopsis alg3* cell culture was composed of the M3 structure which accounted for 51.1–80.1% of total *N*-glycan variants (Figure 8; Table 1; Supplementary Tables 3–6). Plant-specific *N*-glycans were not detected in any of the sites. Moreover, the GAA produced in the NaCl-supplemented culture also showed a similar composition, indicating that increasing the GAA production by NaCl supplementation at a concentration of 50 mM did not affect GAA *N*-glycosylation.

**Table 1:** Composition of the *N*-glycan structures in GAA produced in *Arabidopsis alg3* cell cultures with and without NaCl supplementation.

Abbreviation	Structure	Relative amount (%)									
		95 kDa GAA			76 kDa GAA		95 kDa GAA^NaCl^			76 kDa GAA^NaCl^	
		N390	N470	N882	N390	N470	N390	N470	N882	N390	N470
M1	ManGlcNAc_2_	8.7	1.5	21.2	4.4	1.4	14.7	1.2	33.0	30.1	1.6
M2	Man_2_GlcNAc_2_	6.4	9.5	10.1	11.4	9.5	10.2	7.6	11.3	13.2	11.2
M3	Man_3_GlcNAc_2_	68.0	68.2	51.1	67.5	75.5	56.6	57.8	51.8	54.0	80.1
M4^ER^	Man_4_GlcNAc_2_	–	–	–	2.6	1.8	1.6	1.1	–	0.9	1.1
M5^ER^	Man_5_GlcNAc_2_	–	–	1.5	6.6	4.5	1.4	3.1	–	0.6	2.3
GlcM5^ER^	GlcMan_5_GlcNAc_2_	–	–	–	1.2	2.0	–	2.6	–	–	–
GnM3	GlcNAcMan_3_GlcNAc_2_	12.6	20.0	14.3	3.4	4.0	10.6	25.7	3.9	1.2	3.7
Gn2M3	GlcNAc_2_Man_3_GlcNAc_2_	2.7	0.8	1.8	–	–	2.2	–	–	–	–
GalGnM3	GalGlcNAcMan_3_GlcNAc_2_	1.6	–	–	2.9	1.3	2.0	0.9	–	–	–
GalGn2M3	GalGlcNAc_2_Man_3_GlcNAc_2_	–	–	–	–	–	0.7	–	–	–	–
	Total	100	100	100	100	100	100	100	100	100	100

## Discussion

The first study using an *Arabidopsis alg3* mutant to produce a recombinant protein was reported a decade ago. In this study, a single chain Fv-Fc antibody MBP10 was produced in an *Arabidopsis alg3* mutant plant (Henquet et al., 2011). However, the antibody carried a KDEL ER retention signal so that the antibody accumulated in the ER and consequently bore mostly the aberrant M5 structure, which is unique to the *alg3* mutant, rather than the M3 structure. Since then, no other study has been done using the *alg3* mutant line. In the present study, human recombinant GAA without any ER retention signal was produced in an *Arabidopsis alg3* cell culture, and thereby, the fate of the recombinant protein in the *alg3* mutant cells could be better understood.

GAA is a lysosomal enzyme known to undergo sequential proteolytic processing and *N*-glycosylation for enzyme maturation (Moreland et al., 2012). In humans, GAA is initially synthesized as a polypeptide of 952 amino acids containing an *N*-terminal signal peptide for co-translational transport into the lumen of the ER. The signal peptide is cleaved by the host signal peptidase, generating a 110 kDa GAA precursor. Then, the precursor is cleaved in the *N*-terminus to generate a 95 kDa GAA intermediate. Subsequently, another protease cleaves the *C*-terminus, resulting in a 76 kDa mature GAA. Finally, a protease cleaves the *N*-terminus to generate the final mature GAA of 70 kDa (Moreland et al., 2005). These sequential proteolytic processes are critical for the enzyme activity, as the 76/70 kDa mature forms show 7–10 times greater affinity to the substrate compared to the 110 kDa precursor (Wisselaar et al., 1993; Bijvoet et al., 1998). Yet, the proteases responsible for these processes are still unidentified. In this study, we found that *Arabidopsis alg3* cells were capable of recognizing and cleaving the native human GAA signal peptide in addition to conducting the multiple proteolytic processing, as indicated by the presence of 110, 95, and 76 kDa forms in the GAA-producing cells. However, the 70 kDa form could not be detected, indicating that the essential protease is absent in *Arabidopsis* cells. Moreover, the proportion of the 95 kDa GAA seemed to be higher than that of the 76 kDa GAA. Therefore, to optimize the GAA production in plant culture, it will be necessary to identify the proteases responsible for the cleavages. With this knowledge in hand, it will become possible to increase the production of mature GAA by co-expressing the human protease(s) *in vivo* or by performing the protease cleavage *in vitro*.

Studies on the production of recombinant human GAA in plant cells have been done in tobacco plant (Martiniuk et al., 2013), rice culture (Jung et al., 2016, 2017), and moss culture (Hintze et al., 2020). In all of these studies, GAAs were constructed under plant-derived signal peptides to drive plant organ-specific localization or secretion into the culture medium. In the present study, the native human GAA signal peptide was used. Interestingly, our results showed that *Arabidopsis* cells were capable of recognizing and processing the human GAA signal peptide, as indicated by the detection of a cleaved GAA polypeptide generating the 110 kDa GAA (Figure 1C). This indicates that the human GAA signal peptide was capable of facilitating GAA entrance into the ER, followed by further transport into the Golgi apparatus and, finally, secretion into the plant culture medium.

GAA is known to be extensively *N*-glycosylated—the 95 and 76 kDa forms contain seven and five *N*-glycosylation sites, respectively (Moreland et al., 2005). As expected, the *Arabidopsis alg3*-produced GAAs have a predominantly M3 structure (51.1–80.1%). Moreover, anti-HRP antibody could not detect any plant-specific *N*-glycans in GAA, although a low amount of plant-specific *N*-glycans were detected in the culture (Figure 7). This suggests that the ruling of *N*-glycosylation may be protein specific. The lack of plant-specific *N*-glycans in our GAA would eliminate the concern of potential allergic reaction upon administration to patients with Pompe disease. For further work, it will be preferable to also knock-out the *gnt-I* and *gnt-II* genes in the *Arabidopsis alg3* cell line to increase *N-*glycan homogeneity and ensure the safety of the product. In addition, galactose-terminal *N*-glycans were also detected. This residue is a component of the Lewis a structure commonly found in the *trans* Golgi apparatus and extracellular proteins and is generated by the β1,3-galactosyltransferase enzyme resident in the Golgi apparatus (Fitchette et al., 1999; Strasser et al., 2007). Detection of this structure in GAA confirms that GAA is properly transported to the Golgi apparatus prior to secretion into the plant culture medium.

Previous studies have demonstrated the potentials of some chemicals to improve the production of recombinant proteins in plant culture; however, these effects seem to be dependent on the target protein and plant species. For instance, PVP was reported to enhance the secreted recombinant heavy-chain monoclonal antibody in *Nicotiana tabacum* NT-1 suspension culture (LaCount et al., 1997). On the other hand, Lee et al. did not find any effect of PVP in the production of human granulocyte-macrophage colony-stimulating factor in *N. tabacum* cv. Havana SR1 suspension culture (Lee et al., 2002). Therefore, medium optimization needs to be done for each product and cell line on a case-by-case basis. In this study, a number of chemicals were shown to increase GAA production, including PVP, PEG, NH_4_NO_3_, mannitol, and NaCl. Supplementation with 50 mM NaCl achieved the highest production while maintaining cell growth, resulting in 3.8-fold higher yield than the control.

The increment of GAA production in intra- and extracellular fractions of NaCl-supplemented culture can be attributed to the stabilizing effect of NaCl, which protects GAA from degradation, as indicated by the relative absence of a degraded GAA band as compared to the control (Figure 6B). Moreover, the increased GAA accumulation in the medium of NaCl-supplemented culture might have been the result of enhanced GAA secretion. This hypothesis is based on the previous reports showing that salt (e.g., NaCl) induces the generation of reactive oxygen species which subsequently attacks membrane lipoproteins and causes impaired membrane permeability (Mansour, 2013), thereby facilitating the secretion of intracellular proteins. However, whether this mechanism occurs in our case and contributes to increased GAA secretion is still unknown. Notably, the *N*-glycan profiles of GAAs produced in NaCl-supplemented culture were similar to those in the control, indicating that NaCl supplementation to a final concentration of 50 mM does not affect *N*-glycosylation in the *Arabidopsis* cells. These findings indicate that NaCl supplementation can be an economic, easy, and effective strategy to increase GAA production in the *Arabidopsis* cell culture.

Despite the improved GAA production by NaCl supplementation, the yield was relatively low as compared to the previous studies of GAA production in tobacco seed (40 μg/g; Martiniuk et al., 2013) and rice cell culture (up to 45 mg/l; Jung et al., 2016, 2017). The low level of production could be the result of ER stress as previously observed in *Arabidopsis alg3* plant (Henquet et al., 2008, 2011; Kajiura et al., 2010). For future work, it may be possible to increase GAA production by co-expressing chaperon proteins to alleviate the ER stress (Margolin et al., 2020). Moreover, the GAA production may be improved by combining with other enhanced production of recombinant proteins in plant cells, such as suppression of gene silencing (Papp et al., 2003; Butaye et al., 2004; Jeong et al., 2018), use of more robust expression system (e.g., use of geminiviral replication, double promoter and terminator; Yamamoto et al., 2018), and transient expression strategy (e.g., plant-cell pack technology; Rademacher et al., 2019).

Finally, we succeeded in producing GAA bearing predominantly the M3 structure. Previous studies have highlighted the merit of using M3 for MR-mediated cellular delivery (Van Patten et al., 2007; Shen et al., 2016). Our success in producing GAA with the M3 structure in the *Arabidopsis alg3* cell culture emphasizes the potential of our glycoengineered *Arabidopsis* cell culture for producing other recombinant proteins in which M3 and/or cellular delivery through MR is desired, such as in the enzyme replacement therapies for Gaucher disease, Fabry disease, Wolman disease, and cholesteryl ester storage disease (Du et al., 2005; Limkul et al., 2016; Shen et al., 2016).

## Data Availability Statement

The raw data supporting the conclusions of this article will be made available by the authors, without undue reservation.

## Author Contributions

RS, Florence, HK, and KF conceived and designed the experiments. RS and Florence conducted laboratory experiments and analyses. HK, TO, RM, and KF conducted data analyses as well. RS wrote the manuscript and all authors provided editorial advice and revised manuscript. All authors contributed to the article and approved the submitted version.

### Conflict of Interest

The authors declare that the research was conducted in the absence of any commercial or financial relationships that could be construed as a potential conflict of interest.
