# Supplementary material for: Production of Human Acid-Alpha Glucosidase With a Paucimannose Structure by Glycoengineered Arabidopsis Cell Culture
Source: Front Plant Sci. 2021 Jul 14;12:703020. doi: 10.3389/fpls.2021.703020 (PMC8318038; doi:10.3389/fpls.2021.703020)
Supplement: Supplementary file 1 [file Data_Sheet_1.PDF]

Supplementary Materials

Supplementary Figures

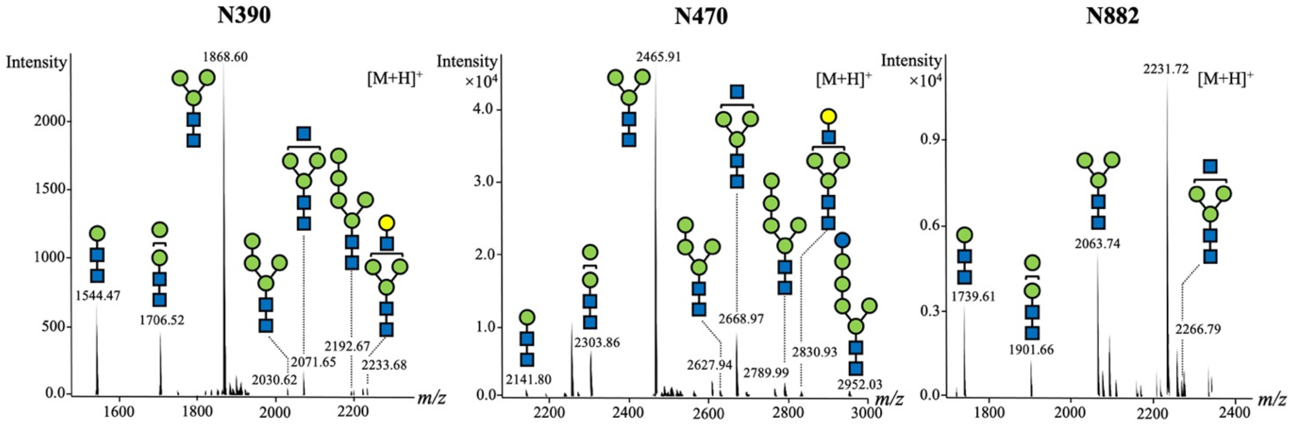

Supplementary Figure 1. Glycoforms of glycopeptides with *N*-glycosylation sites N390, N470, and N882 from the degraded GAA. In the shown deconvoluted MS spectra, the mass $[M+H]^+$ of the tryptic glycopeptides carrying *N*-glycosylation sites (N390, N470, and N882) are depicted.

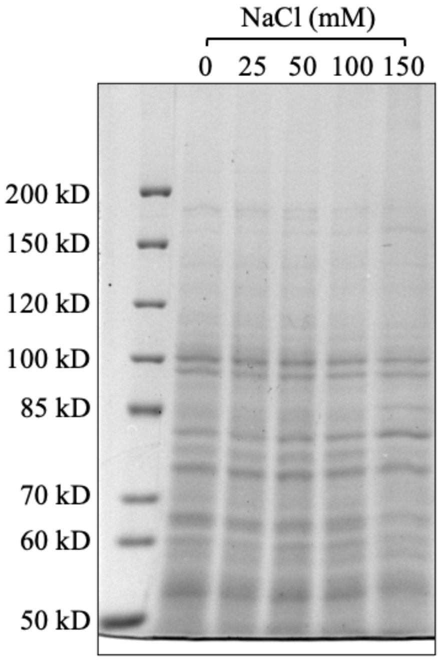

Supplementary Figure 2. CBB staining of intracellular crude protein samples from cultures supplemented with 0-150 mM NaCl, which is used as a loading control for Western blot analysis shown in Fig. 5B.

Supplementary Tables

Supplementary Table 1. Composition of *N*-glycan structures attached on the degraded GAA.

Abbreviation	Structure	Relative amount (%)		
		N390	N470	N882
M1	$ManGlcNAc_2$	16.9	1.6	31.2
M2	$Man_2GlcNAc_2$	11.7	9.9	12.2
M3	$Man_3GlcNAc_2$	62.9	68.4	50.1
$M4^{ER}$	$Man_4GlcNAc_2$	1.5	1.4	-
$M5^{ER}$	$Man_5GlcNAc_2$	0.9	3.0	-
$GlcM5^{ER}$	$GlcMan_5GlcNAc_2$	-	1.2	-
GnM3	$GlcNAcMan_3GlcNAc_2$	4.5	13.5	6.5
GalGnM3	$GalGlcNAcMan_3GlcNAc_2$	1.6	1.0	-
	TOTAL	100	100	100

Supplementary Table 2. GAA purification from the medium of GAA-producing Arabidopsis *alg3* culture.

	Volume (ml)	mg/ml	mg	mU/mg	Total activities (U)	Purification fold	Yield (%)
Crude	210	0.089	18.61	409	7.6	1	100
HIC	15	0.075	1.12	904	1.0	2.2	13.4
SP	0.1	0.18	0.02	10600	0.2	25.9	2.5

Supplementary Table 3. Quantification of *N*-glycan structures on the GAAs produced from Arabidopsis *alg3* cell culture (MS mode).

**Abbreviation**	**Structure**	**Mass-to-charge ratio (*m/z*)**									
		**95 kDa GAA**						**76 kDa GAA**			
		**N390**		**N470**		**N882**		**N390**		**N470**	
		**Theorical**	**Observed**	**Theorical**	**Observed**	**Theorical**	**Observed**	**Theorical**	**Observed**	**Theorical**	**Observed**
Peptide + Gn	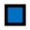	1179.57	1179.50	1776.94	1776.92	1374.72	1374.71	1179.57	1179.45	1776.94	1776.74
Peptide + Gn2	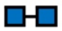	1382.65	1382.60	1980.02	1979.98	1577.80	1577.80	1382.65	1382.43	-	-
Peptide + M1	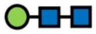	1544.70	1544.63	2142.07	2142.05	1739.85	1739.86	1544.70	1544.51	2142.07	2141.78
Peptide + M2	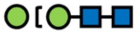	1706.75	1706.68	2304.12	2304.10	1901.90	1901.90	1706.75	1706.51	2304.12	2303.85
Peptide + M3	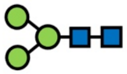	1868.80	1868.73	2466.17	2466.15	2063.95	2063.93	1868.80	1868.59	2466.17	2465.90
Peptide + M4^{ER}	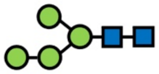	-	-	-	-	-	-	2030.85	2030.60	2628.22	2627.94
Peptide + M5^{ER}	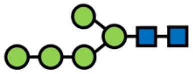	-	-	-	-	2388.05	2388.04	2192.90	2192.67	2790.27	2789.99
Peptide + GlcM5^{ER}	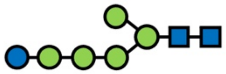	-	-	-	-	-	-	2354.95	2354.77	2952.32	2952.03
Peptide + GnM3	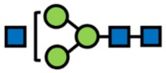	2071.88	2071.79	2669.25	2669.22	2267.03	2267.01	2071.88	2071.63	2669.25	2668.96
Peptide + Gn2M3	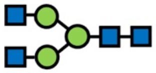	2274.96	2274.87	2872.34	2872.32	2470.11	2470.12	-	-	-	-
Peptide + GalGnM3	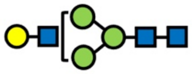	2233.93	2233.89	-	-	-	-	2233.93	2233.64	2831.30	2831.01

Supplementary Table 4. Quantification of *N*-glycan structures on the GAAs produced from Arabidopsis *alg3* cell culture supplemented with 50 mM NaCl (MS mode).

Abbreviation	Structure	Mass-to-charge ratio (*m/z*)									
		95 kDa GAA^{NaCl}						76 kDa GAA^{NaCl}			
		N390		N470		N882		N390		N470	
		Theorical	Observed	Theorical	Observed	Theorical	Observed	Theorical	Observed	Theorical	Observed
Peptide + Gn	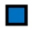	1179.57	1179.42	1776.94	1776.75	1374.72	1374.56	1179.57	1179.41	1776.94	1776.76
Peptide + Gn2	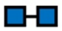	1382.65	1382.45	1980.02	1979.67	1577.80	1577.60	1382.65	1382.45	-	-
Peptide + M1	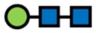	1544.70	1544.49	2142.07	2141.82	1739.85	1739.63	1544.70	1544.50	2142.07	2141.82
Peptide + M2	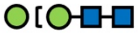	1706.75	1706.55	2304.12	2303.88	1901.90	1901.69	1706.75	1706.55	2304.12	2303.88
Peptide + M3	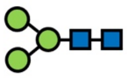	1868.80	1868.61	2466.17	2465.93	2063.95	2063.76	1868.80	1868.62	2466.17	2465.94
Peptide + M4^{ER}	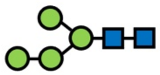	2030.85	2030.58	2628.22	2627.96	-	-	2030.85	2030.65	2628.22	2627.96
Peptide + M5^{ER}	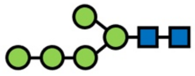	2192.90	2192.70	2790.27	2790.01	-	-	2192.90	2192.70	2790.27	2790.02
Peptide + GlcM5^{ER}	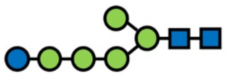	-	-	2952.32	2952.06	-	-	-	-	-	-
Peptide + GnM3	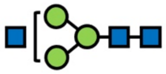	2071.88	2071.63	2669.25	2669.00	2267.03	2266.80	2071.88	2071.63	2669.25	2669.00
Peptide + Gn2M3	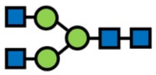	2274.96	2274.72	-	-	-	-	-	-	-	-
Peptide + GalGnM3	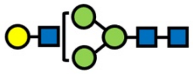	2233.93	2233.67	2831.30	2831.02	-	-	-	-	-	-
Peptide + GalGn2M3	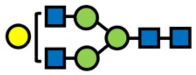	2437.01	2436.76	-	-	-	-	-	-	-	-

Supplementary Table 5. Quantification of *N*-glycan structures on the GAAs produced from Arabidopsis *alg3* cell culture (MS/MS mode).

Abbreviation	Structure	Mass-to-charge ratio (*m/z*)									
		95 kDa GAA						76 kDa GAA			
		N390		N470		N882		N390		N470	
		Theorical	Observed	Theorical	Observed	Theorical	Observed	Theorical	Observed	Theorical	Observed
Peptide + Gn	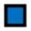	1179.57	1179.51	1776.94	1776.91	1374.72	1374.69	1179.57	1179.45	1776.94	1776.74
Peptide + Gn2	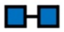	1382.65	1382.57	1980.02	1979.99	1577.80	1577.77	1382.65	1382.51	1980.02	1979.79
Peptide + M1	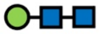	-	-	2142.07	2142.05	-	-	-	-	2142.07	2141.82
Peptide + M2	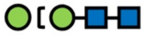	-	-	2304.12	2304.10	-	-	-	-	2304.12	2303.88
Peptide + M3	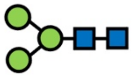	-	-	2466.17	2466.12	-	-	-	-	2466.17	2465.92

Supplementary Table 6. Quantification of *N*-glycan structures on the GAAs produced from Arabidopsis *alg3* cell culture supplemented with 50 mM NaCl (MS/MS mode).

Abbreviation	Structure	Mass-to-charge ratio (*m/z*)									
		95 kDa GAA^{NaCl}						76 kDa GAA^{NaCl}			
		N390		N470		N882		N390		N470	
		Theorical	Observed	Theorical	Observed	Theorical	Observed	Theorical	Observed	Theorical	Observed
Peptide + Gn	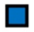	1179.57	1179.45	1776.94	1776.76	1374.72	1374.59	1179.57	1179.42	1776.94	1776.76
Peptide + Gn2	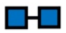	1382.65	1382.48	1980.02	1979.82	1577.80	1577.63	1382.65	1382.45	1980.02	1979.82
Peptide + M1	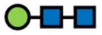	-	-	2142.07	2141.85	1739.85	1739.68	-	-	2142.07	2141.85
Peptide + M2	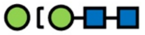	-	-	2304.12	2303.91	1901.90	1901.72	-	-	2304.12	2303.90
Peptide + M3	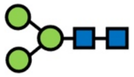	-	-	2466.17	2465.95	2063.95	2063.77	-	-	2466.17	2465.95
